# Monitoring Flower Visitation Networks and Interactions between Pairs of Bumble Bees in a Large Outdoor Flight Cage

**DOI:** 10.1371/journal.pone.0150844

**Published:** 2016-03-16

**Authors:** Mathieu Lihoreau, Lars Chittka, Nigel E. Raine

**Affiliations:** 1 Department of Biological and Experimental Psychology, School of Biological and Chemical Sciences, Queen Mary University of London, Mile End Road, London E1 4NS, United Kingdom; 2 School of Biological Sciences, Royal Holloway University of London, Egham, Surrey TW20 0EX, United Kingdom; University of Northampton, UNITED KINGDOM

## Abstract

Pollinators, such as bees, often develop multi-location routes (traplines) to exploit subsets of flower patches within larger plant populations. How individuals establish such foraging areas in the presence of other foragers is poorly explored. Here we investigated the foraging patterns of pairs of bumble bees (*Bombus terrestris*) released sequentially into an 880m^2^ outdoor flight cage containing 10 feeding stations (artificial flowers). Using motion-sensitive video cameras mounted on flowers, we mapped the flower visitation networks of both foragers, quantified their interactions and compared their foraging success over an entire day. Overall, bees that were released first (residents) travelled 37% faster and collected 77% more nectar, thereby reaching a net energy intake rate 64% higher than bees released second (newcomers). However, this prior-experience advantage decreased as newcomers became familiar with the spatial configuration of the flower array. When both bees visited the same flower simultaneously, the most frequent outcome was for the resident to evict the newcomer. On the rare occasions when newcomers evicted residents, the two bees increased their frequency of return visits to that flower. These competitive interactions led to a significant (if only partial) spatial overlap between the foraging patterns of pairs of bees. While newcomers may initially use social cues (such as olfactory footprints) to exploit flowers used by residents, either because such cues indicate higher rewards and/or safety from predation, residents may attempt to preserve their monopoly over familiar resources through exploitation and interference. We discuss how these interactions may favour spatial partitioning, thereby maximising the foraging efficiency of individuals and colonies.

## Introduction

Understanding how foragers distribute themselves within and among resource patches is a central question in behavioural ecology. Historically it was usually assumed that individuals select and remain in the patches providing them with the highest rewards [1,2]. However, for most animals, foraging decisions are complicated by several additional factors such as an individual’s knowledge of their environment [3], the nutritional composition of foods [4], or their interactions with social partners, competitors and predators [5,6].

Pollinators, for instance, often exploit complex foraging areas comprised of multiple flower patches whose nectar rewards replenish over time [7]. Through repeated visits to familiar places, individuals accumulate knowledge about the location and the profitability of flower patches, enabling them to forage more efficiently than if they explored a novel environment each time [8]. In many species of bees [9,10], butterflies [11], hummingbirds [12] and nectarivorous bats [13], foraging individuals regularly revisit flower patches in stable, repeatable sequences called ‘traplines’. Recent studies on bumble bees collecting sucrose solution from artificial flowers (equivalent, in terms of nectar profitability, to natural flower patches) have begun to reveal how pollinators develop such movement patterns when foraging alone in highly predictable environments, by prioritizing visits to the most rewarding flowers while minimizing overall travel distances between them [14–16]. Whilst this is an important first step, none of these studies have yet captured the considerable additional variation in nectar rewards provided by flowers in field conditions due to the activity of other foragers competing for the same resources [17–21].

Previous attempts to address this question suggest that foragers avoid extensive spatial overlap so that each specializes on a different subset of flowers within larger plant populations. For instance, bumble bees tend to adjust the size of their foraging area in response to changes in the density of conspecific foragers, either by increasing the number of flower patches they visit following the removal of competing foragers [22–24], or by reducing the number of patches they visit after the introduction of new foragers [25]. Similar observations were made with nestmates and non-nestmates, suggesting that bumble bees do not discriminate kin from non-kin during foraging interactions [23,25].

In order to fully understand how these complex patterns of spatial resource partitioning develop over time, as bees learn to exploit their foraging environment, it has now become crucial to study the spatial movements of individual foragers, their behavioural interactions and the potential consequences of such interactions on their future foraging decisions over several consecutive foraging events. In principle, the presence of other foragers can have different effects on a bumble bee’s foraging decisions depending on its experience of the environment. Firstly, foragers can use social information, such as visual or olfactory cues inadvertently provided by conspecifics on flowers, to decide whether or not to visit flowers [26,27]. For instance, inexperienced bees discovering a new foraging environment tend to copy the flower choices of other foragers to identify the most rewarding flowers [28], whereas experienced individuals tend to avoid flowers occupied by conspecifics whose nectar reserves are probably depleted [29]. Secondly, when forager density is high, experienced bees may also try to preserve their foraging area by increasing their visitation rates to particular flowers (exploitative competition [18,21]) or by chasing potential competitors away (interference competition [17,19,20]). Since bumble bees typically enter a foraging environment at different times, depending on their age and the nutritional status of the colony [30], two foragers are unlikely to have the same knowledge about available foraging opportunities. We therefore hypothesize that these natural knowledge asymmetries among foragers have important consequences for their spatial foraging strategies and, ultimately, on resource partitioning.

To explore this possibility, we analysed the foraging patterns of pairs of bumble bees with different levels of experience when exploiting a common array of artificial flowers in a large outdoor flight cage. In each pair, a resident (experienced) forager was allowed to perform 25 foraging bouts before a newcomer (inexperienced) forager was released. Using motion-sensitive cameras mounted on flowers, we mapped the network of flower visitation for each bee, recorded their interactions on flowers and compared their foraging success for another 25 foraging bouts.

## Materials and Methods

**Fig 1:**
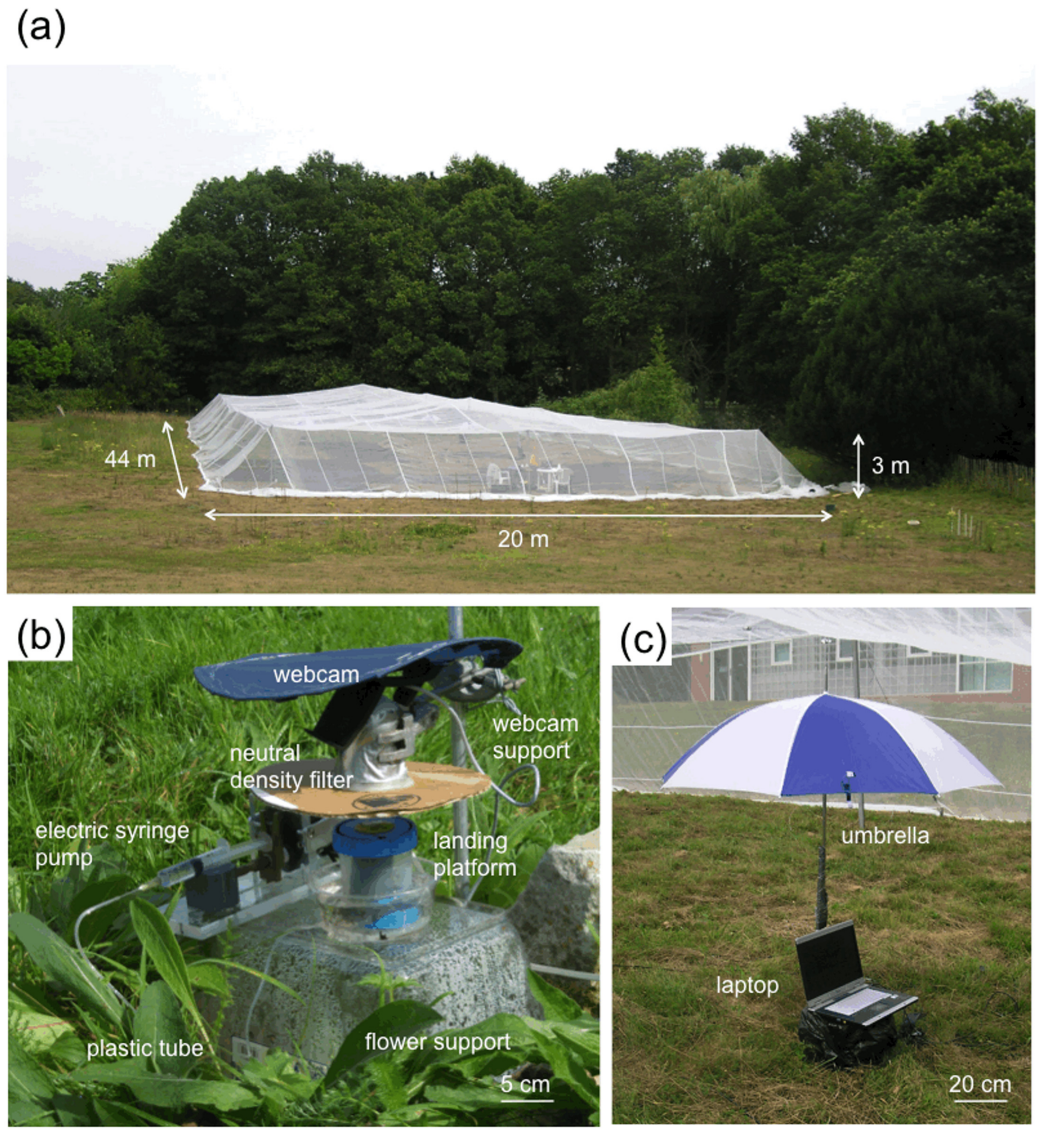
Flight cage, artificial flowers and visual landmarks. (a) The flight cage in the experimental field (Egham, Surrey, UK). (b) Artificial flower consisting of a blue horizontal landing platform, an electric syringe pump, a webcam and their supports (see details in Fig 2a). (c) A laptop protected by a golf umbrella with a unique two-colour pattern acting as a three-dimensional landmark for the bees (photographs by Mathieu Lihoreau).

The study was conducted in August 2010 at the Centre for Agricultural Bioscience International (CABI) centre in Egham (Surrey, UK). The experiments were performed in a large flight cage (length × width × height = 44 × 20 × 3 m, mesh size = 0.5 mm; see Fig 1a) erected on flat pasture. Subjects were workers from a commercially reared *Bombus terrestris* colony (Syngenta Bioline Bees, Weert, The Netherlands). The colony nest box was connected to a transparent Plexiglas entrance tube fitted with a series of shutters to control all bee arrivals and departures. All workers were marked on their thorax with individually numbered tags (Opalith Plättchen, Christian Graze KG, Germany) within a day of emergence from pupae (eclosion). The colony was provided with *ad libitum* defrosted, honeybee-collected, pollen directly into the nest box. Foragers collected sucrose solution (40% v/v) from artificial flowers in the flight cage. All natural flowers inside the cage were removed.

### Artificial flowers and video tracking

**Fig 2:**
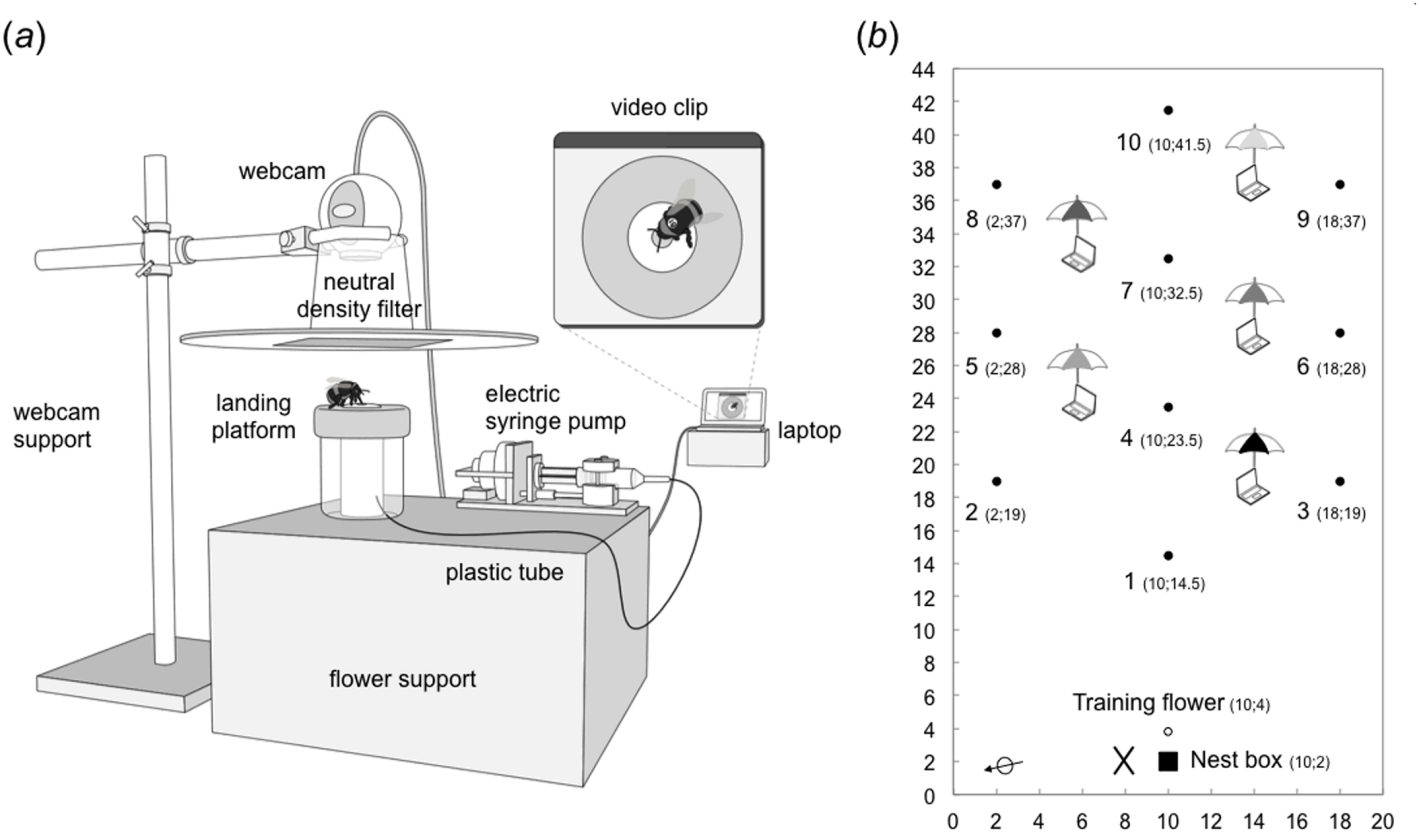
Experimental design. (a) Schematic of an artificial flower and the video tracking system (drawing by Pierre Vedel). As the electric pump depresses the syringe plunger, sucrose solution is pushed through a plastic tube and accumulates at a constant rate (3.3 μL/min) in a feeding cup (capacity = 40 μL) accessed by the bee through a hole in the middle of the horizontal landing platform. A webcam connected to a laptop computer running motion detection software is mounted directly above the landing platform. The webcam was fitted with a neutral density filter (Neutral Density = 0.6) placed on a truncated cardboard cone, to reduce the amount of light entering the lens. Bee movements in the camera field of view trigger recording of a video clip (minimum duration = 5 s), from which the bee’s tag number, its arrival and departure times, and any interactions with another forager can be identified (e.g. S1 and S3 Videos). (b) Spatial arrangement of the nest box, artificial flowers and laptops within the flight cage. Coordinates (x, y) of the nest box (black square), the pre-training flower (open circle) and the test flowers (black circles numbered 1–10) are in metres. Distance between the nest box and the nearest test flower (number 1) was 12.5 m. Distance between any two nearest neighbour test flowers within the array was 9 m (e.g. flowers 1 and 4). Distance between a flower and the nearest laptop was 5.20 m. Each laptop was protected by a golf umbrella, with a unique two-colour pattern, to provide additional three-dimensional landmarks for the bees. The black cross indicates the position of the experimenter. The black arrow (bottom left) indicates north. Photos of the flight cage, the artificial flowers and the visual landmarks are shown in Fig 1.

We used artificial flowers delivering sucrose solution (hereafter ‘nectar’) at a constant rate. Each flower consisted of a landing platform, an electric syringe pump, a webcam and their supports (Figs 1b and 2a). The landing platform was a blue plastic disc (diameter = 60 mm) mounted horizontally on top of a colourless, transparent plastic cylinder (height = 75 mm). The circular shape of the landing platform was reminiscent of many natural flowers commonly visited by bumble bees (e.g. Asteraceae). A small yellow circle (diameter = 20 mm), in the centre of the blue disc, highlighted the location of the feeding cup (capacity = 40 μL) from which bees could collect nectar. The feeding cup was connected to an electric syringe pump (for details see [31]) via a flexible plastic tube (internal diameter = 1 mm, length = 200 mm). As the pump depressed the syringe plunger, nectar was pushed through the plastic tube and accumulated in the feeding cup at a rate of 3.3 μL/min. The landing platform and plastic cylinder sat on a clear plastic support (length × width × height = 300 × 200 × 180 mm) placed on the ground (Figs 1b and 2a). Bees could access the landing platform equally well from all angles. Two bees could extract nectar simultaneously from the same flower (S1 Video).

Bee visits to flowers were recorded automatically using motion sensitive video cameras [32]. A webcam (Logitech c250, Fremont, CA), fitted with a neutral density filter (Neutral Density = 0.6, Lee Filters, Andover, UK) to reduce the amount of light entering the lens, was mounted on top of each flower (Figs 1b and 2a) and powered by a laptop computer running motion detection software (Zone Trigger 2, Omega Unfold, Quebec, Canada). Each webcam recorded a video clip (minimum duration 5 s) every time a bee moved into the camera’s field of view until movement stopped, thus capturing complete flower visits from the point when the bee landed to its departure (e.g. S1, S2 and S3 Videos). The average flower visit duration was 18.77 ± 0.71 s (mean ± standard error (s.e.), n = 5448 visits). Viewing the landing platform from above enabled us to accurately identify bees (from their dorsal numbered tags), their arrival and departure times, and whether they collected nectar from the feeding cup. Video clips in which two bees visited the flower simultaneously also provided data about the nature and frequency of behavioural interactions between foragers.

### Experimental flower array

We used a regular hexagonal array of 10 flowers (Fig 2b), in which nearest neighbour flowers were 9 m apart (e.g. flowers 1 and 4) and second nearest neighbour flowers were 15.8 m apart (e.g. flowers 1 and 6). Testing bees with comparatively few distant artificial flowers increases the probability that they develop efficient foraging routes, reducing travel distances between the different feeding locations, just as they would do between flower patches under natural conditions [14,15,16,32,33]. As bumble bees are unable to detect reflecting (non self-luminant) visual targets presented against a vegetation background subtending a visual angle of *ca*. 3° [34], the maximum distance at which a forager could distinguish a flower (overall dimension: length × width × height = 400 × 300 × 500 mm) from the background is assumed to be 9.6 m. Therefore, it is likely that bees visiting a flower could only detect nearest neighbour flowers in our experimental array.

We used five laptop computers to record the video data. Each laptop ran two webcams and was placed at the centre between three nearest neighbour flowers (distance between the laptop and each of the three flowers = 5.20 m; Fig 2a). Laptops were protected from sun and rain by golf umbrellas (height = 1.5 cm, diameter = 1.0 m). Each umbrella was uniquely identified by a different two-colour pattern and could be used by the bees as a three-dimensional landmark (Fig 1c).

### Experimental procedure

All test bees were nestmates from the same colony. While this might not fully reflect the competitive situations between foragers in the field, there is no evidence that bumble bees recognise nestmates when visiting flowers [23,25]. If we take a colony perspective, when floral nectar is limited, the colony should benefit from all foragers spreading themselves out among flowers, avoiding overlap of foraging areas in just the same way as individuals from different colonies, or indeed members of different species [35,36].

We tested seven pairs of middle-aged foragers (10–15 days post eclosion) to ensure that their age and prior foraging experience was comparable [16]. Bees were pre-trained and tested from 10:00–18:00 on two consecutive days. On day one, we allowed all bees to forage freely on a pre-training flower placed 1 m in front of the nest entrance (Fig 2b) and recorded the identity of regular foragers (bees that were observed visiting the flower and shuttling back and forth between the colony nest box and the flower at least five times within two hours [15]). The pre-training flower was the same shape and colour as test flowers but its feeding cup was filled with a cotton wick dipped into a nectar reservoir from which bees could feed *ad libitum*. During this phase, we covered all test flowers, laptops and umbrellas with black cloth bags so that bees remained naïve to the test situation.

On day two, we tested two randomly selected foragers from the pool of pre-trained bees (from day one) with all test flowers, laptops and umbrellas uncovered. We started the nectar pumps six minutes before the observations to provide 20 μL of nectar in the feeding cup of each flower at the beginning of data collection. A bee visiting the 10 flowers during its first foraging bout could thus gather a minimum of 200 μL of nectar, which is the crop capacity of a large forager [33]. We conducted observations during two consecutive experimental phases, each lasting approximately three hours (mean duration of phase one (± s.e.): 2.5 ± 0.1 h, phase two: 2.9 ± 0.2 h; n = 7 pairs). In the first phase (one-forager phase), we allowed one bee (the resident) to forage for 25 consecutive bouts. A foraging bout started when the forager left the nest and ended upon its return to the colony. In the second phase (two-forager phase), we introduced the second bee (the newcomer) and allowed it to forage alongside the resident. At the start of the two-forager phase the resident bee had 25 bouts experience, while the newcomer had no experience of the flower array. We stopped all observations when the newcomer had completed 25 foraging bouts. Flower visits were automatically recorded using video motion detection and all departures and arrivals at the nest entrance were controlled and timed by the experimenter. Nectar pumps were turned off by the experimenter for flowers that had not been visited by bees for a period exceeding 12 min, in order to cap the maximum standing crop available at flowers to 40 μL. Nectar pumps were restarted immediately after a bee visit. The two experimental phases were conducted in direct succession without interruption so that any sudden change in the foraging pattern of the resident bee between phases would likely be attributable to the release of the newcomer (mean (± s.e.) interval between the last bout of the one-forager phase and the first bout of the two-forager phase: 107.4 ± 14.7 s, n = 7 bouts; mean interval between two consecutive bouts of the same phase: 113.3 ± 7 s, n = 350 bouts). Throughout the observations, the experimenter remained seated next to the colony to control all arrivals and departures of the two tested bees using the shuttered entrance tube of the colony nest box (Fig 2b). At the end of the two-forager phase we cleaned all flowers with a 70% (w/w) ethanol solution. We weighed both foragers and measured their thorax width. Bees from each pair had statistically indistinguishable body mass (mean dry body mass (± s.e.), resident: 133 ± 9 mg, newcomer: 131 ± 6 mg, n = 7 pairs; Wilcoxon signed rank test for paired data: *V* = 16, *P* = 0.813) and body size (mean thorax width (± s.e.), resident: 8.23 ± 0.05 mm, newcomer: 7.98 ± 0.21 mm, n = 7 pairs; Wilcoxon signed rank test for paired data: *V* = 31, *P* = 0.456). All observations were conducted within three weeks on sunny days with a clear blue sky to minimise weather effects as much as possible.

### Data analysis

#### Movement patterns

We recorded 5259 video clips of bees visiting flowers (1763 clips in the one-forager phase, 3496 clips in the two-forager phase). In each clip, we identified the bees’ tag numbers, their time of arrival and departure from the flower, noted whether they fed, and any interactions between foragers. For each bee, we compiled a complete flower visitation sequence from which we constructed a transition matrix containing the cumulative frequency of movements between the nest and each flower, and among all flower pairs, for each experimental phase (for complete list of flower visitation sequences see S1 Table).

**Fig 3:**
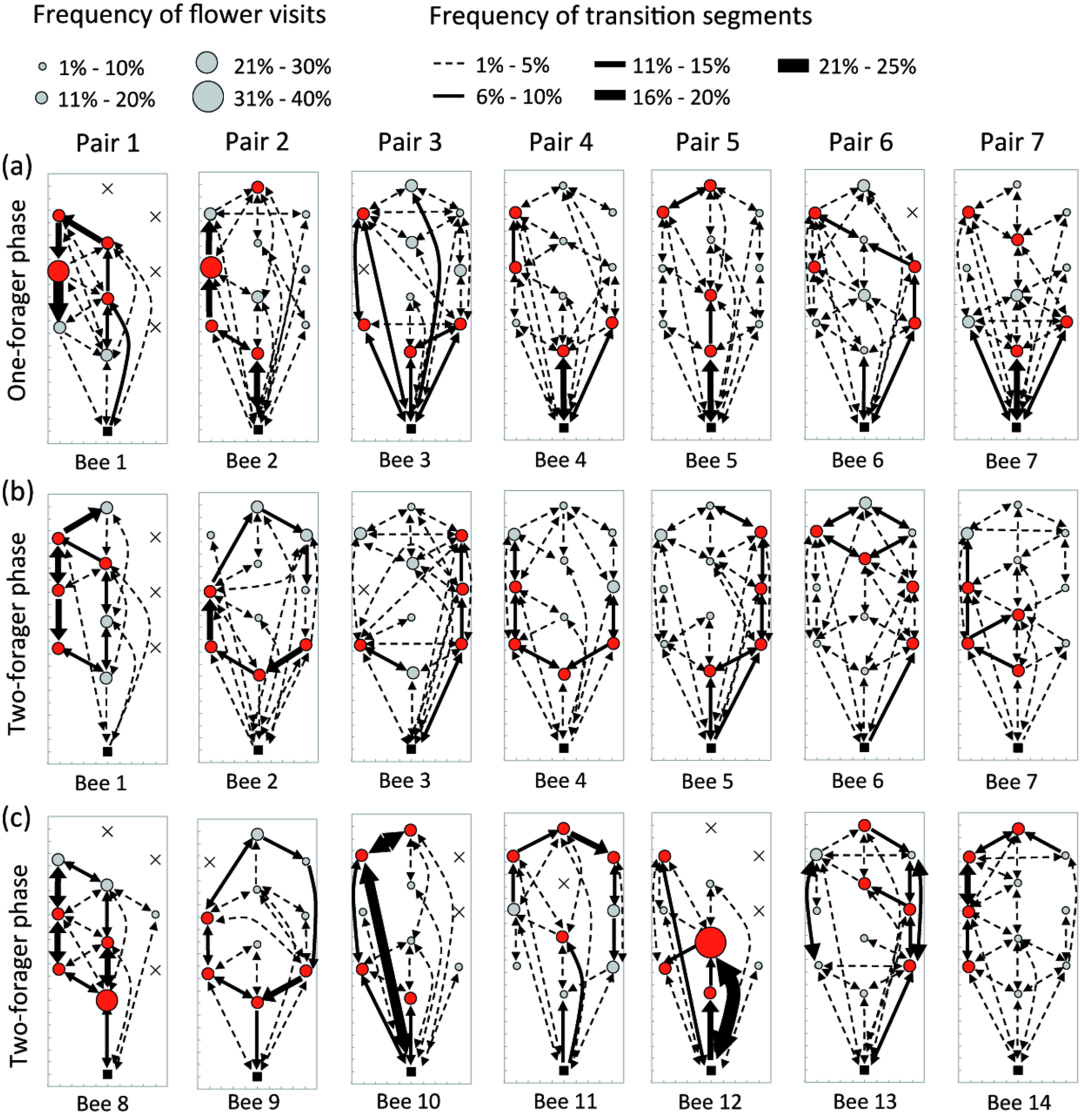
Flower visitation networks. Maps showing the cumulative movement patterns of (a) resident bees during the one-forager phase, (b) resident bees during the two-forager phase, and (c) newcomer bees during the two-forager phase. Visitation networks of bees from the same pair are presented in vertical columns. For each panel, we have represented the nest box (black square), the flowers that were visited at least once (circles), and the flowers that were never visited (grey crosses), by the focal bee. The diameter of each circle is proportional to the cumulative frequency of visits to that flower relative to the total number of visits to all flowers. The four flowers visited most frequently by the focal bee are shown in red. Arrows indicate the frequency and direction in which the bee moved between pairs of locations. Arrow thickness is proportional to the cumulative frequency of transitions between locations relative to the total number of transitions observed. Single-headed arrows indicate the bee was significantly more likely to move in one direction during that transition (binomial test with probability 0.5 to move in either direction, *P* < 0.05). Two-headed arrows indicate symmetrical transition direction. Labels (Bee 1–14, Pair 1–7) refer to the same individuals throughout the study.

From these transition matrices we mapped the movement patterns of individual bees in the form of a weighted, directed visitation network [37], in which the nest and the flowers are nodes and the transitions between nodes are edges (Fig 3). Node diameter and edge thickness arrows are proportional to their relative usage frequency. Directional transitions made more frequently than expected by chance (binomial test with probability 0.5 to move in either direction, *P* < 0.05) are represented with a single-headed arrow. Transitions with no significant directional bias are indicated by two-headed arrows.

From the flower visitation sequences we calculated the number of different flowers visited and the number of revisits to the same flower for each foraging bout. We estimated the minimum overall travel distance (by adding all linear distances between consecutive flower visits, starting and ending at the nest) and the travel speed (overall distance travelled divided by travel duration) for every foraging bout. We quantified similarity between pairs of flower visitation sequences using a similarity index derived from DNA sequence alignment [33,37]. This index takes into account the length of visitation sequences, the identity of flowers visited and visit order. It ranges between 0 (completely different sequences, e.g. 123 vs 456) and 1 (identical sequences, e.g. 12345 vs 12345).

#### Foraging success

For each bee, we calculated the bout duration (time between departure from and arrival at the nest), the total time spent visiting flowers, and the total time spent travelling among flowers for each foraging bout. We estimated the volume of nectar collected during each flower visit, assuming that: (1) nectar accumulated at a constant rate (3.3 μL/min) until the maximum standing crop (40 μL) was reached, (2) all the nectar accumulated prior to and during a visit was removed by a bee, and (3) half of the nectar was taken by each bee if the pair fed simultaneously on the same flower (video data do not allow any more precise estimation of volumes collected). Using the total volume of nectar collected (by summing the nectar volume collected for all flower visits) per foraging bout we calculated the net energy intake rate (*E*), an established metric to estimate bumble bee foraging success [38–41], using the equation:

$$E\;=\;\frac{epSV-M(c_{p}T_{p}\;{+\;c}_{f}T_{f}}{T_{p}{+\;T}_{f}}$$

where *e* is the energy contained in 1 mg of sucrose (15.48 J), *p* is nectar density (1.177 mg/μL at 20°C), *S* is nectar concentration (40% v/v), *V* is the volume of nectar collected (μL), *M* is forager mass (g), *c*_*p*_ is the energetic cost of probing (0.034 J/g; [42]), *T*_*p*_ is the probe duration (time spent visiting flowers), *c*_*f*_ is the energetic cost of flight (0.436 J/g [42]) and *T*_*f*_ is the flight duration (time spent travelling among flowers).

#### Interactions on flowers

Pairs of bees were observed on the same flower in 189 of the 5259 (3.6%) video clips. We identified five types of behavioural events involving the two foragers in these clips: following (one bee landed on a flower less than 5 s after the other bee departed; S2 Video); joining (one bee landed on an already occupied flower; S1 and S3 Videos); feeding simultaneously (two bees collected nectar simultaneously on the same flower; S1 Video); pushing (one bee pushed the other bee with its head or legs; S3 Video); and eviction (one bee moved away, or fell off, the landing platform immediately after being pushed; S3 Video). Joining, feeding simultaneously, pushing and eviction were independent events that could occasionally, but not always, be observed in a sequence within the same video clip (see for examples in S1 and S3 Videos). Behaviours of the same type separated by at least 3 s were treated as different events [43]. Bees were never observed to bite or sting each other.

#### Statistical analyses

All data were analysed in R 3.1.2 [44]. We used Fisher’s exact tests to assess whether bees visited individual flowers at similar rates. We conducted Mantel tests and Pearson’s correlation coefficient (function mantel.rtest() in R package ‘ade4’ [45]) for pairwise comparisons of transition matrices for the same bees during the two experiment phases (one- and two-forager), and for different bees during the same experimental phase. We used Generalized Linear Mixed Models (GLMMs; function glmmPQL() in R package ‘MASS’ [46]) to analyse data on foraging success and interactions on flowers, while accounting for repeated measures. We used GLMMs with Gaussian errors for analyses of nectar volumes collected, foraging bout durations, travel speeds, net energy intake rates and Mantel correlation coefficients (*r*). We used GLMMs with Poisson errors for analyses of frequencies of flower visits and interactions on flowers. Behavioural comparisons of the same individual across multiple foraging bouts (either within or between experimental phases) included bee identity as a random factor. Comparisons between bees within the same pair, or between bees from different pairs, included pair identity as a random factor. We used Wilcoxon signed rank tests for paired samples to compare both similarity indices for the same bee at different stages of the experiment and visit frequencies to preferred flowers between bees within the same pair. All means are given with standard errors (mean ± s.e.).

## Results

### One-forager phase

#### Movement patterns

Each of the seven bees tested discovered the flowers sequentially in a different order (S1 Table). After 25 foraging bouts, bees had discovered an average of 9.14 ± 0.55 flowers (n = 7 bees): four bees found all ten flowers, two bees found nine flowers and one bee found six (Fig 3a). Overall flower visitation frequencies were significantly different among bees (Fisher’s exact test: *P* < 0.001), indicating that each forager explored and used the experimental array in a different manner. On average, bees made 60.4 ± 2.7% (n = 7 bees) of all their visits to only four flowers and these subsets were unique to each forager (see red circles in Fig 3a). This suggests that there was no flower, or subset of flowers, that was inherently more attractive to all bees than others. As bees gained experience with the spatial configuration of flowers, their visitation sequences from successive bouts increased in similarity, from an average similarity index of 0.25 ± 0.04 in the first 5 bouts to 0.36 ± 0.06 in the last 5 bouts (n = 7 bees; Wilcoxon test: *V* = 0, *P* = 0.016). Therefore, in the absence of other foragers, resident bees developed a foraging area in which they restricted their foraging activity to a subset of flowers that they visited in an increasingly repeatable order.

#### Foraging success

**Fig 4:**
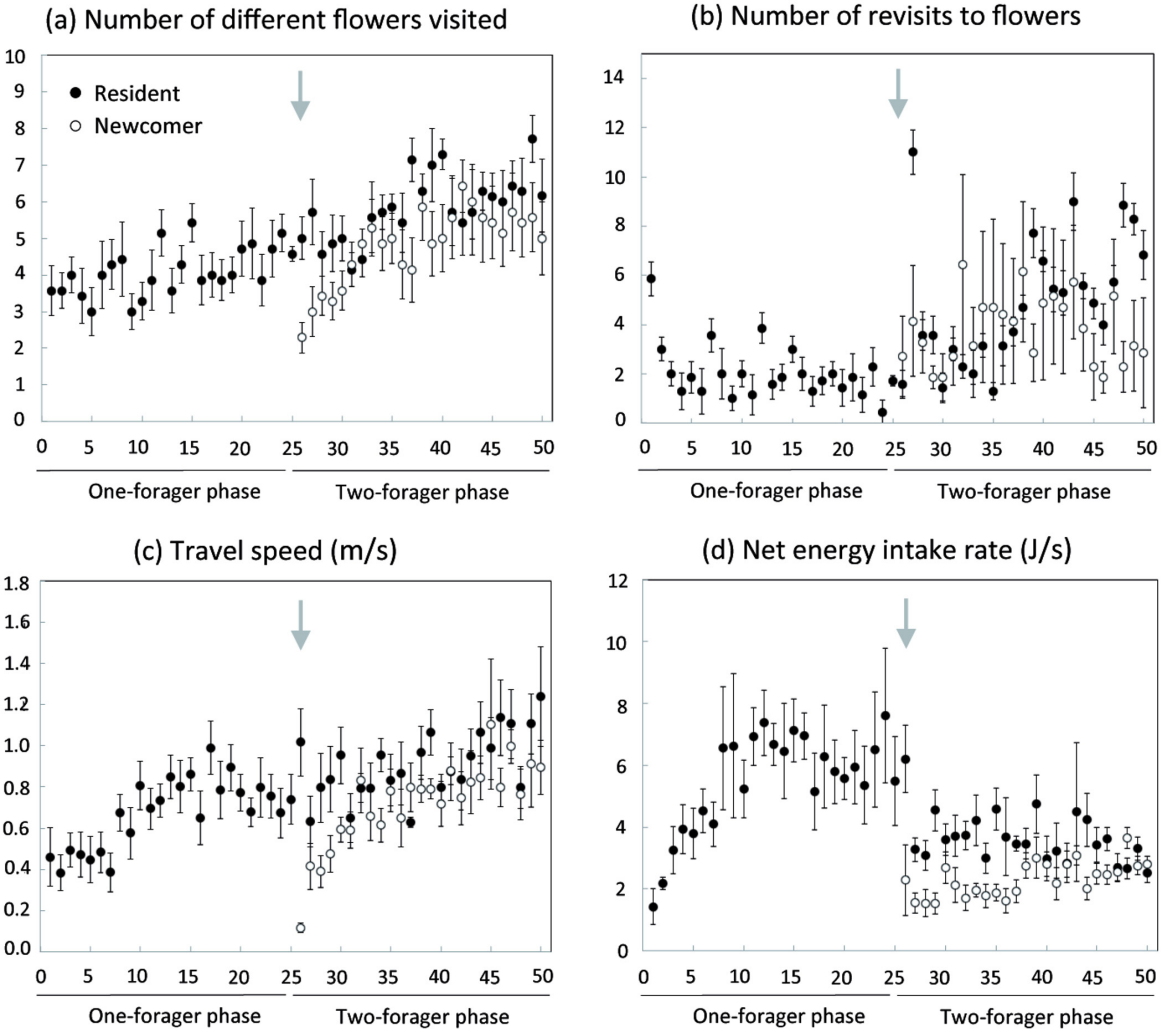
Foraging success. Average data (mean ± s.e.) for the seven resident (black dots) and the seven newcomer (white dots) bees during all consecutive foraging bouts of the two experimental phases. (a) Mean number of different flowers visited per foraging bout; (b) number of revisits to the same flowers per foraging bout; (c) travel speed per foraging bout; (d) net energy intake rate per foraging bout. Grey arrows show the moment when newcomers were introduced in the array of flowers (bout 26). Additional foraging bouts by resident bees during the two-forager phase (> bout 50) are not shown.

Each bee collected a consistent volume of nectar per foraging bout throughout the 25 bouts (first 5 bouts: 169.3 ± 30.7 μL, last 5 bouts: 176 ± 29.6 μL, n = 7 bees; GLMM—effect of number of bouts completed: *t*_6_ = 0.18, *P* = 0.860). This suggests that bees filled their crop to capacity in all foraging bouts. However, their foraging success improved dramatically with training. As bees gained experience, they performed significantly shorter foraging bouts (first 5 bouts: 475.7 ± 107.6 s, last 5 bouts: 217.8 ± 17.9 s, n = 7 bees; GLMM—effect of number of bouts completed: *t*_6_ = -2.53, *P* = 0.045), visited a greater number of flowers per bout (first 5 bouts: 3.51 ± 0.18, last 5 bouts: 4.63 ± 0.46, n = 7 bees, GLMM—effect of number of bouts completed: *t*_6_ = -2.93, *P* = 0.026; Fig 4a), made fewer revisits to the same flowers per bout (first 5 bouts: 2.8 ± 0.57, last 5 bouts: 1.2 ± 0.51, n = 7 bees, GLMM—effect of number of bouts completed: *t*_6_ = -2.90, *P* = 0.027; Fig 4b) and increased their travel speed per bout (first 5 bouts: 0.45 ± 0.73 m/s, last 5 bouts: 0.73 ± 0.07 m/s, n = 7 bees, GLMM—effect of number of bouts completed: *t*_6_ = 3.1, *P* = 0.021; Fig 4c). Ultimately, bees improved their net energy intake rate by 128 ± 54% (first 5 bouts: 2.91 ± 0.23 J/s, last 5 bouts: 6.18 ± 0.99 J/s, n = 7 bees; GLMM—effect of number of bouts completed: *t*_6_ = 3.43, *P* = 0.014; Fig 4d). All these improvements in measures of foraging performance had levelled off by the end of the 25 bouts (Fig 4).

### Two-forager phase

#### Movement patterns

Immediately after the resident bee completed 25 foraging bouts, we released a second forager (newcomer) into the flight cage and recorded all flower visits made by both bees until the newcomer had also completed 25 bouts (S1 Table). For each resident, networks of flower visits during the one-forager and two-forager phases were significantly correlated (Fig 3b; Mantel test; bee 1: *r* = 0.79, *P* < 0.001; bee 2: *r* = 0.39, *P* = 0.001; bee 3: *r* = 0.47, *P* = 0.002; bee 4: *r* = 0.65, *P* < 0.001; bee 5: *r* = 0.57, *P* < 0.001; bee 6: *r* = 0.59, *P* < 0.001; bee 7: *r* = 0.29, *P* = 0.032).

Despite this general similarity in resident bee movement patterns during the two phases of the experiment, some remarkable changes were also observed. Firstly, resident bees made 83.4 ± 1.8% (n = 7 bees) more flower visits per foraging bout during the two-forager phase compared to the one-forager phase (mean number of visits per bout during the one-forager phase: 6.14 ± 0.98, two-forager phase: 10.66 ± 1.72, n = 7 bees; GLMM—effect of experiment phase: *t*_6_ = 3.48, *P* = 0.013). Secondly, each resident continued to make most of its visits to a subset of four flowers (57.1 ± 1.8% of all visits, n = 7 bees), but on average 1.43 ± 0.3 of those flowers were different from those visited most frequently by the same bee during the one-forager phase (see red circles in Fig 3a and 3b). Thus, although each individual retained the core structure of its foraging area during the two-forager phase, the arrival of a newcomer affected the size and geometry of its foraging area.

On average, newcomers discovered 8.57 ± 0.48 flowers (n = 7 bees) during the two-forager phase, creating appreciable potential for interference with movement patterns of the resident bees. Three bees found all 10 flowers, one bee found nine flowers, one bee found eight flowers and two bees found seven (Fig 3c). Newcomers and residents from the same pair never used exactly the same visitation sequence (S1 Table), but their overall flower visitation networks were significantly correlated for six of the seven bee pairs (Mantel test; pair 1: *r* = 0.74, *P* < 0.001; pair 2: *r* = 0.89, *P* = 0.001; pair 3: *r* = 0.32, *P* = 0.042; pair 4: *r* = 0.52, *P* < 0.001; pair 5: *r* = 0.19, *P* = 0.114; pair 6: *r* = 0.77, *P* < 0.001; pair 7: *r* = 0.78, *P* = 0.032). The newcomer from pair 5 (bee 12) exhibited remarkably little exploratory behaviour: it made the fewest flower visits (n = 61 visits) and the highest proportion of visits to the same flower (34.4% of visits to flower 4) of all 14 bees tested. Importantly, flower visitation networks of newcomers were significantly more correlated to the visitation networks of residents from their own pair than to the visitation networks of residents from any other pair (*r* same pair: 0.59 ± 0.10, n = 7 correlations, *r* different pair: 0.35 ± 0.03, n = 42 correlations; GLMM—effect of same or different pair: *t*_6_ = 3.06, *P* = 0.022). Thus, the foraging choices of bees in each pair influenced each other and these individuals developed movement patterns that were significantly more similar than expected by chance. Like residents, newcomers also made most of their visits to a subset of only four flowers (64.8 ± 4.3% of all visits, n = 7 bees), and this subset was unique to each individual (see red circles in Fig 3c). The overall frequency of visits to the subset of four flowers during the two-forager phase was similar in residents and newcomers (Wilcoxon signed rank test: *V* = 3, *P* = 0.078). On average, 1.86 ± 0.51 (n = 7 bees) of the four flowers most frequently visited by a newcomer were the same flowers visited most frequently by the resident from the same pair, thus confirming that bees exploited foraging areas with considerable spatial and resource use overlap.

#### Foraging success

Resident bees completed a greater number of foraging bouts (residents: 28.7 ± 1.2 bouts, newcomers: 25 bouts; n = 7 bees; Wilcoxon signed rank test: *V* = 26.5, *P* = 0.042) and collected more nectar (residents: 3.76 ± 0.28 mL, newcomers: 2.28 ± 0.36 mL; n = 7 bees; Wilcoxon signed rank test: *V* = 28, *P* = 0.016) than newcomer bees during the two-forager phase. However, comparing flower visitation sequences of residents immediately before and after the beginning of the two-forager phase reveals a sharp drop in foraging success associated with the release of the newcomer. Residents made longer foraging bouts (last 5 bouts of one-forager phase: 206.3 ± 9.5 s, first 5 bouts of two-forager phase: 293.5 ± 28 s, n = 7 bees; GLMM—effect of number of bouts completed: *t*_6_ = -3.14, *P* = 0.02), made more flower revisits (last 5 bouts of one-forager phase: 1.2 ± 0.57, first 5 bouts of two-forager phase: 4.23 ± 1.37, n = 7 bees; GLMM—effect of number of bouts completed: *t*_6_ = -2.73, *P* = 0.034; Fig 4b) and had lower net energy intake rates per bout (last 5 bouts of one-forager phase: 6.18 ± 0.99 J/s, first 5 bouts of two-forager phase: 4.14 ± 0.37 J/s, n = 7 bees; GLMM—effect of number of bouts completed: *t*_6_ = 2.88, *P* = 0.028; Fig 4d) during the first 5 bouts of the two-forager phase than during the last 5 bouts of the one-forager phase. These measures of the foraging success for residents remained stable until the end of the two-forager phase (GLMM—effect of number of bouts completed: *P* < 0.05 for all three variables, Fig 4). The foraging success of residents showed markedly different dynamics during the one-forager phase (a gradual increase followed by a stabilisation) compared to the two-forager phase (sharp drop followed by a stabilisation), thus illustrating the influence of newcomers on resident behaviour.

In contrast, newcomer mean foraging success improved throughout the two-forager phase. These bees, with no previous experience of the flower array gradually reduced the duration of their foraging bouts (first 5 bouts: 432.3 ± 50.1 s, last 5 bouts: 249.2 ± 17.7; s, n = 7 bees; GLMM—effect of number of bouts completed: *t*_6_ = -3.37, *P* = 0.015), while increasing the number of different flowers they visited per bout (first 5 bouts: 3.11 ± 0.41, last 5 bouts: 5.37 ± 0.85 n = 7 bees; GLMM—effect of number of bouts completed: *t*_6_ = 3.93, *P* = 0.007; Fig 4a) and their travel speed per bout (first 5 bouts: 0.4 ± 0.05 m/s, last 5 bouts: 0.87 ± 0.07 m/s, n = 7 bees; GLMM—effect of number of bouts completed: *t*_6_ = 6, *P* = 0.001; Fig 4c). Ultimately, newcomers reached similar levels of route repeatability (mean similarity index for residents: 0.35 ± 0.03, newcomers: 0.44 ± 0.07; Wilcoxon signed rank test: *V* = 7, *P* = 0.297) and net energy intake rates (residents: 3.35 ± 0.18 J/s, newcomers: 2.84 ± 0.11 J/s; GLMM—effect of bee status: *t*_6_ = -1.93, *P* = 0.101; Fig 4d) as residents during the last 5 bouts of the two-forager phase. Therefore the resident competitive advantage, attributable to experience accumulated during the one-forager phase, progressively disappeared as newcomers became increasingly familiar with the spatial arrangement of flowers.

Comparing the overall foraging efficiency of newcomers during the two-forager phase to that of residents during the one-forager phase indicates that the gradual improvement of foraging performances in newcomers was limited, to some extent, by the presence of residents. Newcomers made foraging bouts of similar duration (newcomers: 294.8 ± 23.4 s, residents: 280.4 ± 22.2 s, n = 7 bees; GLMM—effect of bee status: *t*_6_ = 0.55, *P* = 0.6), had similar travel speeds (newcomers: 0.72 ± 0.07 m/s, residents: 0.72 ± 0.06 m/s, n = 7 pairs; GLMM—effect of bee status: *t*_6_ = 0.07, *P* = 0.948), visited similar numbers of flowers per bout (newcomers: 4.81 ± 0.66, residents: 4.05 ± 0.31, n = 7 pairs; GLMM—effect of bee status: *t*_6_ = 0.78, *P* = 0.468) and made similar numbers of flower revisits per bout (newcomers: 3.8 ± 1.31, residents: 2.09 ± 0.76, n = 7 pairs; GLMM—effect of bee status: *t*_6_ = -1.2, *P* = 0.274) compared to residents during the one forager-phase. However, energy intake rates of newcomers remained significantly lower than those of residents during all foraging bouts of the two-forager phase (newcomers: 2.45 ± 0.23 J/s, residents: 5.52 ± 0.43 J/s, n = 7 pairs; GLMM—effect of bee status: *t*_6_ = -6.71, *P* < 0.001), highlighting the negative impact of competitive interactions on the ability of newcomers to efficiently establish and exploit their own foraging area.

#### Interactions on flowers

**Fig 5:**
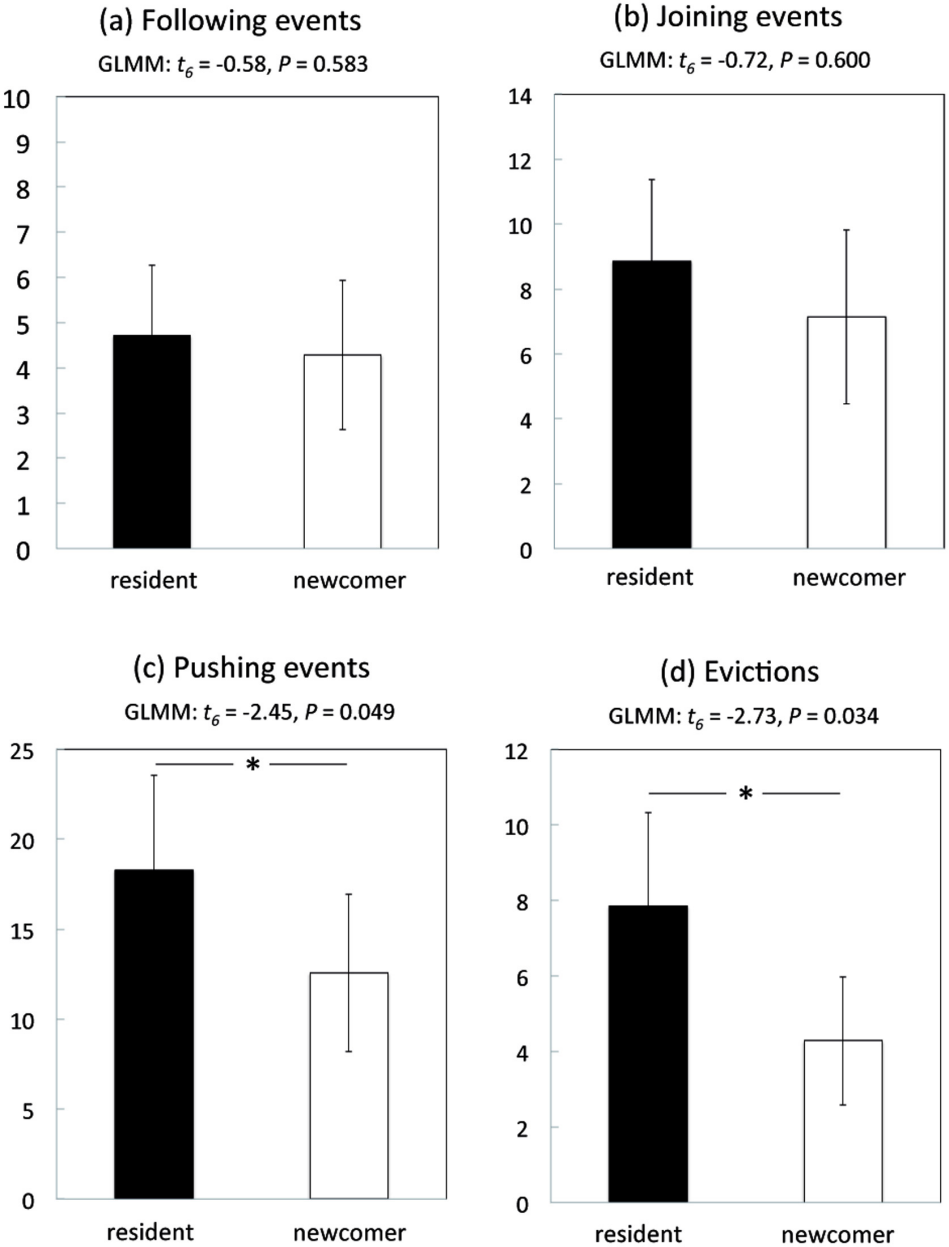
Interactions on flowers. Cumulative data (mean ± s.e.) for the seven resident (black bars) and the seven newcomer (white bars) bees showing: (a) total number of following events (one bee landed on a flower less than 5 s after the other bee departed) (b) total number of joining events (one bee landed on an already occupied flower); (c) total number of pushing events (one bee pushed the other bee with its head or legs); (d) total number of evictions (one bee moved away, or fell off, the landing platform immediately after being pushed). For each pairwise comparison, the results of a GLMM are shown (Poisson GLMM, random effect: pair identity, fixed effect: bee status (resident or newcomer) on variable of interest). Asterisks represent significant differences (*P* < 0.05).

Video recording of all flower visits provided us with critical information on the frequency and outcomes of interactions between foragers. Bees followed each other on flowers (one bee landed on a flower after the other departed; S2 Video) in 1.8% of all visits (63 of the 3496 clips in the two-forager phase). Bees joined each other on flowers (one bee landed on an already occupied flower; S1 and S3 Videos) in a further 3.6% of all visits (126 of 3496 clips). These direct encounters on flowers occurred in all pairs and their frequency tripled over time, from an average of 2.00 ± 0.87 encounters during the first 5 bouts of the two-forager phase to 7.14 ± 2.71 during the last 5 bouts (GLMM—effect of number of bouts completed by the newcomer: t_6_ = 2.58, *P* = 0.042). Nonetheless, the relative rarity of these events indicates that bees did not deliberately move behind one another most of the time. While residents and newcomers both followed (GLMM—effect of bee status: t_6_ = -0.58, *P* = 0.583; Fig 5a) and joined (GLMM—effect of bee status: t_6_ = -0.72, *P* = 0.6; Fig 5b) each other on flowers at similar rates overall, newcomers were never observed to follow or join residents on their first visit to each of the 10 flowers. This initial absence of choice copying by newcomers at previously unvisited flower locations suggests that these bees gained sufficient experience with the phenotype of our artificial flowers (colour, shape, texture) during the pre-training phase to consider it familiar.

The two bees fed simultaneously on the same flower in only 6.3% of all visits with an encounter (8 of 126 clips; S1 Video). The other 93.7% of visits with an encounter (118 of 126 clips) were characterized by physical interactions during which bees pushed each other on the landing platform. In 28.8% of these interactions (34 of 118 clips), both foragers left the flower simultaneously without feeding. In the other 71.2% (84 of 118 clips), one bee evicted the other from the feeding platform. Bees initiated pushing at similar rates irrespective of whether the encounter occurred on one of their four most visited flowers or not (GLMM—effect of flower type (one of the four most favoured flowers or not): t_11_ = 0.98, *P* = 0.349; effect of bee status: t_5_ = 1.2, *P* = 0.285; interaction: t_11_ = -1.69, *P* = 0.118). However, resident foragers initiated 64.4 ± 7.0% of all pushing events (GLMM—effect of bee status: t_6_ = -2.45, *P* = 0.049; Fig 5c) and provoked 68.9 ± 6.9% of all evictions (GLMM—effect of bee status: t_6_ = -2.73, *P* = 0.034; Fig 5d), thus dominating overall interactions on flowers. Presumably, residents had greater motivation to escalate aggressive interactions than newcomers due to their prior experience and greater knowledge of the flower reward values.

**Fig 6:**
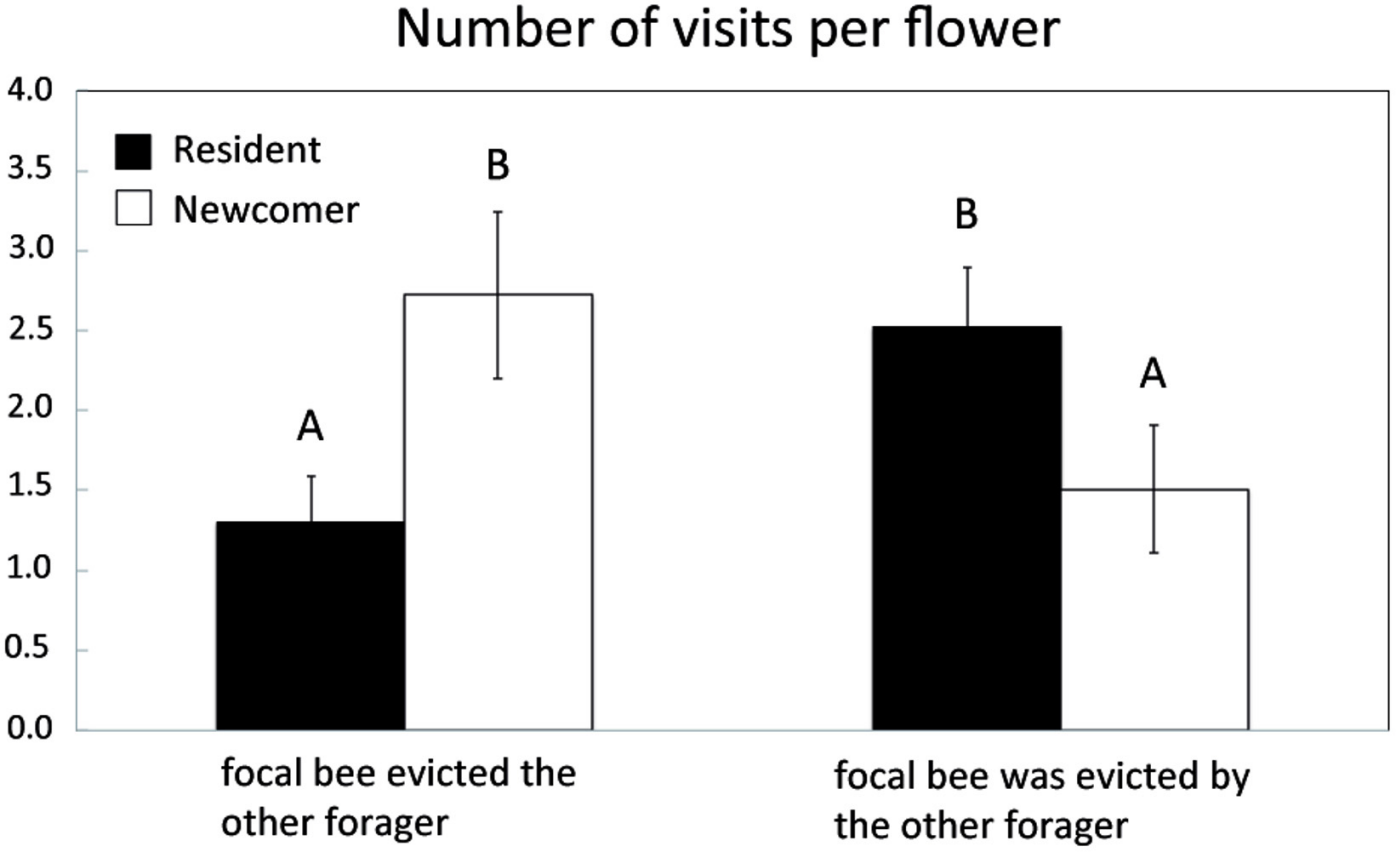
Consequences of direct encounters on subsequent flower visits. Average (mean ± s.e.) number of visits to a flower during a foraging bout in relation to the outcome of encounters on that flower in the previous bout for the seven resident and the seven newcomer bees: either the focal bee evicted the other forager or the focal bee was evicted by the other forager. Data shown are for resident (black bars) and newcomer (white bars) bees. Upper case letters represent significant differences (*P* < 0.05; Poisson GLMM, random effects: flower and pair identities, fixed effect: outcome of eviction from a flower in the previous bout on the number of visits to that same flower in the current bout).

Although relatively infrequent, these interactions on flowers were correlated with subsequent changes in bee foraging decisions. Both residents and newcomers showed significantly increased visit frequencies to the flower(s) on which they had encountered each other during the preceding bout (GLMM—effect of encounter frequency at a flower during previous bout: t_68_ = 3.55, *P* < 0.001; effect of bee status: t_6_ = -2.08, *P* = 0.083, interaction: t_68_ = 0.05, *P* = 0.962). However, they exhibited distinct responses depending on the outcome of previous encounters (Fig 6). Resident foragers prioritized visits to flowers from which they had been evicted, possibly to monopolize familiar nectar sources in their foraging area. In contrast, newcomers made a greater number of visits to flowers from which they had evicted residents. By prioritizing visits to flowers on which they displaced the resident, and thus obtained a food reward, newcomers could be attempting to establish their own foraging area. Ultimately, both residents and newcomers increased their visits to the same individual flowers, thus increasing the chances of encounters with each other and spatial overlap between their respective foraging areas.

## Discussion

Bumble bees foraging simultaneously in a common environment adopted different strategies depending on their experience of the flower array. Residents, that had started to establish a foraging area several hours before the arrival of newcomers, continued to exploit familiar feeding sites by increasing their frequency of floral visits and evicting newcomers when they encountered them on flowers. In contrast, newcomers prioritized revisits to flowers from which they had successfully evicted residents and obtained a nectar reward, presumably to establish their own foraging area. Our results highlight significant spatial overlap between bee foraging areas, which may have emerged from this combination of exploitation and interference.

In the absence of other foragers, bumble bees given exclusive access to multiple replenishing feeding sites tend to exploit a subset of these resources within larger foraging areas based on their spatial memories [10]. Over consecutive bouts, bees develop routes (traplines) enabling them to adjust their timing of revisits to feeding sites to enable nectar replenishment [14], prioritize visits to most rewarding sites [15] and minimize overall distances travelled between them [16,31]. Consistent with findings from a previous study on a different bumble bee species (*Bombus impatiens*) [24], we found that foraging experience confers a competitive advantage to bees; enabling experienced residents to visit more feeding sites, travel faster between them, and achieve greater foraging success than less well informed newcomers. Our analyses of individual movement patterns show how this home advantage diminishes with time, due to a sharp drop of the foraging success of residents, that suddenly lose their exclusive access to resources, and a gradual increase of newcomer success, as they discover flowers and start to include them in their developing foraging areas.

Several lines of evidence indicate that the behavioural changes of residents are a direct consequence of the introduction of newcomers. Firstly, immediately following the start of the two-forager phase (bouts 26–30), resident bees made 42% longer foraging bouts, 253% more revisits to empty flowers per bout, resulting in 33% lower energy intake rates than when they foraged alone. The sharp drop in resident foraging performance at the start of the two-forager phase, followed by a stabilisation of foraging success shows markedly different dynamics to the typical gradual increase and stabilisation of foraging success observed in bees foraging alone in stable arrays of flowers (as described in the one-forager phase and in numerous other studies using similar experimental approaches [7,14–16,24,25,32,33]). Secondly, the sudden changes in resident foraging behaviour were accompanied by a significant shift in both the size and geometry of their foraging area. Bees with exclusive access to a stable array of flowers establish durable traplines to exploit selected feeding sites as long as these sites continue to provide enough resources [7,14,16]. When the array is perturbed, for instance because some flowers are experimentally moved [32,33], their relative rewards are changed [15], or competitors are added or removed [22–25], bees search for new feeding sites and modify the their established foraging areas. Therefore, sudden alterations of resident behaviour between the two experimental phases observed in our study cannot be explained by them simply accumulating more foraging experience, but are instead the result of the presence of newcomers.

Interestingly, the foraging areas of residents and newcomers showed significant levels of spatial overlap notwithstanding that there were enough resources available for foragers to exploit different subsets of flowers. Spatial overlaps are not the consequence of random movements since flower visitation patterns were more similar between foragers within a pair than those of different pairs. The fact that resident bees continuously revisited the same flowers/areas throughout the two experimental phases also indicates that environmental heterogeneities within the flight cage (e.g. light, temperature, humidity, wind), which may have greatly fluctuated between the start (morning) and the end (evening) of the observations, had no apparent influence on space use by bees. Furthermore, we found no indication that any flower positions were more attractive than others as all pairs of bees tested focused their foraging areas around different subsets of flowers. Spatial overlaps are also unlikely to have emerged from a tendency for bees to follow one other, as foragers followed and joined each other on the same flowers in less than 6% of all recorded visits. Instead, our data show that bees engaged in competitive interactions over access to flowers.

Video recording of all flower visits revealed that more than 90% of encounters on flowers involved physical interactions, during which bees pushed each other, resulting in the eviction of one contestant. In contrast to the overt aggressive events observed among bumble bee workers competing over reproduction in colonies that have passed the ‘competition point’ [43], interactions on flowers did not result in visible injuries or death, suggesting that bees attempted to monopolize nectar rewards rather than directly impair the long-term performance of a potential rival forager. Aggressive interactions on flowers have been previously reported between different bee species competing for limited nectar or pollen resources, for instance in bumble bees [17] and stingless bees [20]. However, we are not aware of previous reports about such interactions between conspecifics. Nieh [47] described aggressive interactions between honeybees (*Apis mellifera*) on abundant food resources that could accommodate up to 40 bees, an experimental context that was probably closer to hive robbing (when stronger colonies attack weaker hives to steal their honey stockpiles) than flower foraging. This explanation is unlikely for our experiments as they involved low forager densities and multiple flowers each providing small amounts of nectar when compared to *ad libitum* feeding conditions [47].

Although relatively rare (less than 4% of all flower visits), these encounters on flowers may have had critical consequences for bees’ subsequent foraging decisions, depending on their individual experience. Resident foragers initiated and won most interactions, indicating that they engaged in active defence of their foraging area. Such ‘prior-residence effect’ (*sensu* [48]) on contest outcomes suggests that resident bees had a stronger motivation to contest and escalate competition due to the greater value they placed on these resources that are part of their foraging area, compared to less experienced newcomers [49]. In the rare cases when newcomers won interactions on flowers, residents increased their visitation rates to contested flowers in the subsequent foraging bout, potentially an attempt to discourage newcomers from revisiting flowers in future bouts by keeping nectar rewards low [12]. In contrast, newcomers increased their visitation rates to flowers from which they recently evicted residents. Presumably, the nectar rewards obtained after a successful eviction of residents reinforced newcomer motivation to exploit particular flowers and make it more likely they are included in their developing foraging area or trapline through simple associative learning [50].

Although our study involved interactions between nestmates, we are not aware of any evidence that bumble bees respond differently to nestmates compared to other conspecifics in a foraging context. Several recent studies indicate that bees equally use social information when deciding to either join or avoid conspecifics on flowers [26,27,28,29], be they closely related nestmates or foragers from different colonies, thus suggesting that similar results would be observed with foragers from different colonies. Moreover, if bees could recognise non-nestmates, we would expect competitive interactions to be even more pronounced in a situation involving multiple colonies. Nonetheless it would be useful to conduct similar experiments with pairs of workers from different colonies to explore whether the levels of interference competition and aggression depend on relatedness between foragers.

It is likely that these competitive interactions on flowers were favoured (at least initially) by cues inadvertently provided by both competitors. Although we found no indication that bees visually followed one another to choose the same flowers, they had access to olfactory footprint cues that accumulated on visited flowers throughout the experiment [26]. Bees learn to associate these scent marks with reward levels experienced on flowers and develop different responses depending on context, so that the same mark can become attractant, neutral or repellent to a forager based on its past foraging success [51]. Since all foragers tested in our study were pre-trained in groups on a single flower delivering *ad libitum* nectar rewards before being tested, it is likely that they each associated the presence of scent marks (due to repeated visits by multiple bees from the colony) with flowers containing high rewards in areas relatively safe from predators. During the two-forager phase, newcomers may thus have initially been attracted to flowers already exploited and scent marked by residents, increasing the probability of encounters on flowers. However, after a few foraging bouts each bee may have adjusted its interpretation of scent marks based on individual experience of rewards from flowers.

Overall our results suggest that the development of bumble bee foraging areas occurs through a combination of resource depletion and interference. These mechanisms have been proposed to explain resource partitioning among territorial animals competing for divisible spaces, such as large habitat patches [52,53]. Future studies on pollinator space use might be expanded to explore this hypothesis in experimental scenarios involving more foragers and from more colonies in various arrangements of flowers. Specifically, the frequency of the competitive interactions between foragers in the field, where nectar secretion rates of individual flowers are typically lower ([54–56] but see [57]) and the number of available flowers per bee may be larger than in our experimental conditions, remain to be confirmed. Potential behavioural differences among foragers from different colonies will also have to be examined [58,59]. How, or indeed whether, foraging interactions and their consequences on space use observed in pairs of bees might scale up to the level of colonies, populations and communities, and how they could shape patterns of pollination are exciting questions deserving future attention.

## Supporting Information

S1 Table Complete list of flower visitation sequences compiled from video clips recorded at each flower. For each bee, the visitation sequence from each consecutive foraging bout is presented in chronological order down a column. Numbers (1–10) refer to flower locations (Fig 2b), labels (Bee 1–14, Pair 1–7) refer to the same individuals throughout the study, and an empty cell indicates that a bee was not allowed to forage during that bout.
(DOCX)

S1 Video Joining event followed by simultaneously feeding. A bee (bee 10, tag: white 99) lands on a flower already occupied by another bee (bee 3, tag: white 88). The two bees extract nectar simultaneously (for *ca*. 9 s) and leave the flower when the cup is emptied. Labels (bee 3 and 10) refer to the same individuals throughout the study.
(M4V)

S2 Video Following event. A bee (bee 1, tag: white 32) lands on a flower 2 s after a previous bee (bee 8, tag: white 24) has left. Bee 8 probes the empty feeding cup and leaves. Labels (bee 1 and 8) refer to the same individuals throughout the study.
(M4V)

S3 Video Joining event followed by a pushing event and an eviction. A bee (bee 6, tag: white 32) lands on a flower already occupied by another bee (bee 13, tag: white 24). Bee 6 pushes bee 13 in the back and evicts it from the landing platform. Bee 6 probes the empty feeding cup, then leaves. Labels (bee 6 and 13) refer to the same individuals throughout the study.
(M4V)
