# Supplementary material for: Monitoring Flower Visitation Networks and Interactions between Pairs of Bumble Bees in a Large Outdoor Flight Cage
Source: PLoS One. 2016 Mar 16;11(3):e0150844. doi: 10.1371/journal.pone.0150844 (PMC4794241; doi:10.1371/journal.pone.0150844)
Supplement: S1 Table — For each bee, the visitation sequence from each consecutive foraging bout is presented in chronological order down a column. Numbers (1–10) refer to flower locations (Fig 2b), labels (Bee 1–14, Pair 1–7) refer to the same individuals throughout the study, and an empty cell indicates that a bee was not allowed to forage during that bout. (DOCX) [file pone.0150844.s001.docx]

|  |  | Pair 1 | | Pair 2 |  | Pair 3 |  | Pair 4 |  | Pair 5 |  | Pair 6 |  | Pair 7 |  |
| --- | --- | --- | --- | --- | --- | --- | --- | --- | --- | --- | --- | --- | --- | --- | --- |
| Test phase | Bout | Resident  (Bee 1) | Newcomer  (Bee 8) | Resident (Bee 2) | Newcomer  (Bee 9) | Resident (Bee 3) | Newcomer  (Bee 10) | Resident (Bee 4) | Newcomer  (Bee 11) | Resident (Bee 5) | Newcomer  (Bee 12) | Resident (Bee 6) | Newcomer  (Bee 13) | Resident (Bee 7) | Newcomer  (Bee 14) |
| One-forager  phase | 1 | 55522222 |  | 558106 |  | 103333139101010 |  | 1111445458 |  | 1473145510212410731458 |  | 1118 |  | 77778878 |  |
|  | 2 | 888555552 |  | 89 |  | 333699910 |  | 1144588741 |  | 147810 |  | 8858 |  | 7810107755 |  |
|  | 3 | 4485441 |  | 58910 |  | 669998888 |  | 76143 |  | 46774 |  | 458105510221 |  | 58 |  |
|  | 4 | 881010104111552 |  | 94 |  | 221 |  | 3 |  | 1148 |  | 5107213 |  | 87442 |  |
|  | 5 | 41114775 |  | 33 |  | 88791088 |  | 18 |  | 31 |  | 3334 |  | 2381022762 |  |
|  | 6 | 4877185552 |  | 3378521 |  | 8810107333 |  | 31 |  | 14587699 |  | 148 |  | 1 |  |
|  | 7 | 888521152222447885 |  | 33779521 |  | 8763 |  | 631 |  | 363699109610961 |  | 1344478 |  | 112 |  |
|  | 8 | 42 |  | 222211 |  | 87311 |  | 458716810933 |  | 710969939105 |  | 45810742 |  | 1114 |  |
|  | 9 | 45522 |  | 6455 |  | 13339 |  | 8 |  | 12 |  | 33458 |  | 1141082 |  |
|  | 10 | 44552245258 |  | 11145 |  | 344 |  | 910 |  | 35 |  | 3458436 |  | 22117108 |  |
|  | 11 | 145852447 |  | 111225 |  | 132 |  | 367581031 |  | 48 |  | 3678106 |  | 3 |  |
|  | 12 | 477885522255222552525258741 |  | 1125582 |  | 114882 |  | 367852 |  | 138 |  | 36581016782 |  | 37642 |  |
|  | 13 | 478522578441 |  | 11222258 |  | 28 |  | 31 |  | 125 |  | 3672 |  | 478211 |  |
|  | 14 | 441114525584117 |  | 2558107 |  | 13 |  | 4521 |  | 14710 |  | 36786106 |  | 34764 |  |
|  | 15 | 4785211478852 |  | 14582 |  | 287 |  | 3367525810312161081 |  | 125810341 |  | 366425 |  | 11337991082 |  |
|  | 16 | 477178552147852 |  | 125885211 |  | 731 |  | 258 |  | 1258 |  | 367852 |  | 13 |  |
|  | 17 | 47858552147852 |  | 1425810 |  | 2810 |  | 1125 |  | 14 |  | 310521 |  | 137 |  |
|  | 18 | 752257478741785 |  | 3675585 |  | 39 |  | 458 |  | 178 |  | 8424510 |  | 476 |  |
|  | 19 | 785214717852244 |  | 14510 |  | 33982 |  | 42221 |  | 48106 |  | 678104 |  | 1313 |  |
|  | 20 | 458717878452 |  | 255103 |  | 14 |  | 312 |  | 14581079 |  | 11525810631 |  | 1379 |  |
|  | 21 | 478525874111114 |  | 251041 |  | 2879 |  | 555 |  | 4810107312581081041109 |  | 368521 |  | 61088 |  |
|  | 22 | 478522147852 |  | 258104 |  | 23 |  | 58101 |  | 7107 |  | 3672810 |  | 322 |  |
|  | 23 | 478552147887418522 |  | 25104 |  | 14428 |  | 5810 |  | 34478109611 |  | 367521 |  | 114 |  |
|  | 24 | 4785211 |  | 2581041 |  | 3810 |  | 1 |  | 46910852 |  | 145874 |  | 3479 |  |
|  | 25 | 7821852 |  | 15104 |  | 2233822810 |  | 331 |  | 42352231 |  | 367610 |  | 2 |  |
| Two-foragers phase | 26 | 78521478521 | 1 | 109 | 331111 | 91085 | 55555555555881010 | 1096312106 | 473 | 4791041 | 1 | 1810296 | 258866 | 147783775 | 366 |
|  | 27 | 478525258741752581 | 155884 | 113 | 35555555555555555522 | 142763 | 88101010 | 310821 | 555 | 379108 | 4 | 1478103 | 8961 | 45823345824107510109641258974114785521691092181094341082391085410851 | 339910852233 |
|  | 28 | 4752178521785211475 | 155 | 510 | 445552257 | 33992213 | 101082221 | 58109631 | 588 | 142 | 144 | 361088233 | 8510962289636369931 | 69102113 | 458991010 |
|  | 29 | 785210 | 1111 | 3455 | 55101071 | 9972 | 81010108 | 425810858213 | 58101 | 36782 | 1142 | 34810943671096282 | 310698236647 | 275 | 336910 |
|  | 30 | 7887410 | 4457 | 410 | 45101052 | 71076312 | 810 | 312103125 | 45810 | 3642691082 | 42 | 3679108 | 3678967826788 | 48741 | 1081010852 |
|  | 31 | 7478527810 | 14588521 | 22510 | 710105555233710431 | 426997 | 8101010822 | 68521 | 448810 | 378 | 14 | 1342 | 35896 | 925 | 39108552 |
|  | 32 | 7855224 | 1114445884478852228222211144852 | 5109 | 710633111310105 | 6631369 | 8102111 | 3116312 | 458101 | 136798 | 147 | 3679108 | 3671072 | 3455 | 3910851023910910855 |
|  | 33 | 4781041475 | 111458855211114142 | 31 | 331367105 | 3699236 | 810882 | 42587631 | 4109331052 | 1428109631 | 14 | 76106413781081062 | 3678109 | 1134 | 3691085524452 |
|  | 34 | 7852225810 | 155241147852222522241175521 | 12510109 | 7109931337551031 | 23369783631 | 2821 | 33645555 | 45109 | 382139 | 14 | 367109421063 | 678412810 | 146425589641 | 391085411 |
|  | 35 | 77528101041 | 14225411125787411787122222111147 | 391021 | 10931 | 69821 | 810777 | 3691052 | 410931091010 | 1985231 | 158 | 342879910852 | 3471096 | 1425771 | 42573491085 |
|  | 36 | 478101 | 22588552241114412252252521 | 367109996335106631 | 7 | 63910821 | 8104441 | 364510106311 | 451063 | 7 | 1 | 342258 | 3671097691081 | 342579521534552 | 124910852589 |
|  | 37 | 7581071 | 187752222581147852258741 | 3745109663312555555 | 1411 | 691097721 | 14 | 31223642552510961 | 441093 | 13452 | 45 | 3489621 | 781096 | 12581094169721431 | 491085258109852113 |
|  | 38 | 78521478521 | 1444255252211221452587876 | 55555551069331 | 147552137494111 | 3798216972 | 72 | 2510731 | 458109632 | 1452 | 8247 | 1376910821336782 | 33771063 | 257413884521775821 | 24585109631124810963139105 |
|  | 39 | 758104145225810852221 | 14521125574 | 455546333377910551251096311 | 1953123653142 | 97213641 | 18 | 36225 | 4582523697 | 369963 | 58 | 14910821378109681 | 4231 | 314764225109641342581099642 | 378521258579 |
|  | 40 | 7858101078552810104411147810852852 | 1458522158747855221177522142 | 410910513367 | 37109631 | 342810963 | 1411 | 3258107312516 | 691084 | 34251096 | 14 | 3691028761258109618511 | 810963 | 24741145841 | 42589742137109108541 |
|  | 41 | 781047118101085252108858852582141 | 14145587875522125877744121 | 2510 | 3677222136451091 | 72369 | 88 | 21258 | 488109631 | 34251096 | 48 | 888716788 | 367821 | 1342581097411258109642 | 758109810521397521 |
|  | 42 | 8525810744155221258744111447810 | 14212586885587101055222155858106 | 425 | 67710931125109631 | 312 | 66422 | 45214810631 | 558109631 | 36763 | 581074 | 4791021 | 2810963 | 14225734258104213421 | 24375212589 |
|  | 43 | 8108108108552525810810525888585211414752 | 14785441178582211 | 2593125963137521259105211 | 74251096312510931213579722 | 67810 | 31 | 36 | 469108541 | 27 | 4584 | 781096113678107631 | 346710963 | 241143785213691085241 | 258107241132584191085 |
|  | 44 | 7810521147785810104711211478105 | 48521 | 37952313442596312575213 | 36752510931122551041 | 3677269 | 8 | 36910852 | 369107 | 3694 | 422 | 378521 | 63118109631710632 | 4258109421 | 4281085413791085248105 |
|  | 45 | 7852581010744411781074147810822 | 114776 | 274251010 | 7109312510963 | 981093910 | 8104 | 4587312 | 109631 | 78211 | 48 | 4781096 | 143281074 | 42581097521342581074 | 9108213864581091085211 |
|  | 46 | 281081010741147852581074 | 141476 | 2510963125109631 | 137109312513 | 721 | 42114 | 42258107 | 458109631 | 369 | 4228 | 378109742 | 769 | 3475213125810942 | 7521258107143 |
|  | 47 | 7810274125810744177871010104112 | 414147106 | 25109105213451031 | 9105521367710312593125610931 | 2610 | 33 | 52113108521 | 81096312581096331 | 36593146 | 287 | 17910852 | 71094128 | 142581096421342582 | 28741342581074311 |
|  | 48 | 4755810810107417107417781074111214752125875 | 4763 | 251096312510963125510631211 | 2510144293 | 8107 | 8107 | 1422861072 | 81010963142581093 | 3109 | 42 | 347852136 | 3678212810 | 134258974125881097421 | 7852114758852 |
|  | 49 | 78107108552552118108107521 | 4763 | 2510931331471055213669710 | 3125931142510931 | 429108 | 32 | 142810763155810796312 | 458109631 | 16910851399101096391 | 47 | 17910858796 | 8212810 | 42897413148255109 | 325810741375521345874 |
|  | 50 | 7810852581081074411252581074 | 413 | 109312551094 | 35214311342510109312510931 | 710 | 82 | 431228943128107 | 1458109631 | 2631 | 44258 |  | 121097125810 | 342589761258974213697521 | 587 |
|  | 51 | 55788522122585258525810 |  | 251096331259 |  | 369781061 |  | 4581076312125787 |  | 69108221 |  |  |  | 342581096125871 |  |
|  | 52 | 478525212581081074 |  | 2510963121371052510312510312594 |  |  |  |  |  | 1369108 |  |  |  | 342378321258109642 |  |
|  | 53 | 7810108521475 |  | 255710931225 |  |  |  |  |  | 3127 |  |  |  | 4342581099742581096 |  |
|  | 54 | 7781074112252581074 |  | 92523155109105213143910510741 |  |  |  |  |  |  |  |  |  | 42581097413125897421 |  |
|  | 55 | 7855222178585211225810744 |  | 4259311225109312596331257521311459741 |  |  |  |  |  |  |  |  |  | 342589752131228109 |  |
|  | 56 |  |  |  |  |  |  |  |  |  |  |  |  | 3477896421133421 |  |
|  | 57 |  |  |  |  |  |  |  |  |  |  |  |  | 3425810997 |  |
|  | 58 |  |  |  |  |  |  |  |  |  |  |  |  | 134258 |  |
|  | 59 |  |  |  |  |  |  |  |  |  |  |  |  | 45897521 |  |
